# Supplementary probiotic complexity modulates growth performance, physiological status, and water quality of super-intensive Pacific white shrimp (*Litopenaeus vannamei*) biofloc system

**DOI:** 10.3389/fmicb.2026.1924931

**Published:** 2026-09-17

**Authors:** Xiaohan Li, Jingyi Zhang, Hongwei Wu, Buwen Sun, Jiarui Liu, Nasi Xiao, Huan Xiang, Haiyan Liu, Qingyang Li, Zhi Li

**Affiliations:** 1 Hebei Key Laboratory of Animal Physiology, Biochemistry, and Molecular Biology, Hebei Collaborative Innovation Center for Eco-Environment, College of Life Sciences, Hebei Normal University, Shijiazhuang, China; 2 Ministry of Education Key Laboratory of Molecular and Cellular Biology, Shijiazhuang, China; 3 Laboratory of Dietary Component Interactions and Precision Nutrition, Hebei Agricultural University, Baoding, China; 4 AquaOne Tangshan Aqua-Tech Co., Tangshan, China; 5 South China Sea Fisheries Research Institute, Chinese Academy of Fishery Sciences, Beijing, China

**Keywords:** biofloc technology, growth performance, immunity, probiotic consortia, water quality

## Abstract

Supplementing biofloc systems with functionally specialized probiotics represents a promising strategy for super-intensive Pacific white shrimp (*Litopenaeus vannamei*) aquaculture. Nevertheless, the efficacy and the microbial mechanism are still opaque. Based on heterotrophic nitrifying bacterium *Providencia rettgeri* (N), which has exhibited holistically promoting effects to the shrimp biofloc system, we further compared the impacts (N) with a dual consortium (N + *Bacillus subtilis*; NB) and a triple consortium (NB + effective microorganisms; NBE). Our results demonstrated that survival rates from all groups exceeded 76% with no significant differences. Regarding growth performance, NB simultaneously promoted the growth speed and the feed utilization of shrimp, whilst NBE exerted negative effects on feed efficiency. Besides, NB consortium kept the lowest inorganic nitrogen in the rearing water. Microbiome profiling of biofloc revealed NB induced a distinct microbial structure, characterized by nitrifying bacteria (e.g., *Nitrosomonas*) and intestinal symbionts (e.g., *Candidatus Bacilloplasma*). Physiologically, shrimp reared in NB showed higher catalase activity. In contrast, NBE suppressed superoxide dismutase activity and induced compensatory transcription of digestion (*trypsin* and *lipase*) and lipid metabolism (*peroxisome proliferator-activated receptor γ*). Transcriptomic analyses further elucidated, in comparison with N, NB stimulated host energy metabolism via the TCA cycle and glycolysis, whereas NBE triggered cellular stress and macromolecular catabolism, notably enriching autophagy and ubiquitin-mediated proteolysis pathways. In conclusion, the combination of *P. rettgeri* and *B. subtilis* synergistically optimizes the output and ecology to the super-intensive *L. vannamei* biofloc system. Excessive microbial complexity results in a metabolic burden, forcing the host to prioritize stress responses instead of growth. Further work should concentrate on optimizing probiotic formulas in which prioritize ecological compatibility over mere strain richness.

## 1 Introduction

Pacific white shrimp (*Litopenaeus vannamei*) is one of the most economically important species in global aquaculture, contributing the largest share of crustacean production worldwide (FAO, 2024). To satisfy the increasing market demand and compensate for declining wild shrimp stocks, super-intensive farming has become the mainstream farming model (Villarreal and Juarez, 2022; Ciji and Akhtar, 2021; Boyd et al., 2020). However, such intensive culturing systems inevitably result in severe environmental and health challenges, particularly the rapid accumulation of toxic nitrogenous wastes and, consequently, devastating outbreaks of *Vibrio* pathogens (Krummenauer et al., 2011; Emerenciano et al., 2022; Aguilera-Rivera et al., 2019). Historically, antimicrobials have been extensively used as a panacea for prophylactic, therapeutic, and growth-promoting effects in shrimp aquaculture (Lulijwa et al., 2020). However, with the global banning of antibiotic growth promoters due to the rise of antimicrobial resistance, the industry urgently seeks effective alternatives that are not only environmentally sustainable but also economically viable (Thornber et al., 2019; Cabello et al., 2013; Defoirdt et al., 2007; Boyd et al., 2020).

Among these sustainable strategies, biofloc technology (BFT) has emerged as a highly promising, multifaceted alternative to antimicrobials (Crab et al., 2012; Xu et al., 2013). Conventionally, by manipulating the carbon-to-nitrogen ratio (C/N), BFT drives a sophisticated consortium comprising heterotrophic microorganisms, which assimilate inorganic nitrogen into microbial biomass, and chemoautotrophic bacteria, which facilitate nitrification (Ebeling et al., 2006; Pérez-Fuentes et al., 2016). These microbial communities aggregate into biofloc particles, thereby enabling *in situ* nitrogen recycling and serving as a continuous probiotic reservoir for the host (Avnimelech, 1999; Ahmad et al., 2017). Nevertheless, the traditional BFT relying on the spontaneous development of indigenous microbial communities, often exhibits severe functional instability and ecological fluctuations across different batches (Ray et al., 2010; Khanjani and Sharifinia, 2020; Li et al., 2024b). This unpredictability clouds the prospect of BFT and highlights the necessity for a more stable and targeted interventional strategy.

To overcome the instability of traditional BFT, “supplementary biofloc” characterized by continuous addition of microorganisms has been recently introduced as a targeted microbial modulation strategy (Silva et al., 2022; de Souza Valente et al., 2020). Our previous work pioneered the application of *Providencia rettgeri* in shrimp BFT, demonstrating for the first time its exceptional holistic effects, while simultaneously validating the growth-promoting effects of *Bacillus subtilis* (Li et al., 2024a).

The current studies in shrimp supplementary biofloc rearing systems mostly focus on the impacts of a single species or a genus (Wu et al., 2022; Puvanasundram et al., 2021). There is very limited information on systematic effects of co-supplementation of microbiologically distinct taxa with complementary obligate advantages. Moreover, the ecological and metabolic consequences of increasing microbial complexity remain unexplored, particularly when systems transiting from a single baseline strain to more complex consortia. Therefore, though theoretically available, it remains unclear whether and how these distinct functional groups collaborate to influence the biofloc microbiome, the water environment, and modulate host physiological responses.

To explore the potential of multi-probiotic supplementary strategy, our study evaluated the impacts of progressively complex microbial interventions on the super-intensive *L. vannamei* biofloc system. Using *P. rettgeri* as the baseline, we incorporated *B. subtilis*, capable of secreting extracellular enzymes and antimicrobial compounds, to create a dual-probiotic consortium (Panigrahi et al., 2022; Kuebutornye et al., 2019). Furthermore, we added a commercial effective microorganism (EM) mixture to this dual-probiotic combination and built a more complex consortium. We hypothesized that these assembled microbiomes would synergistically drive efficient nitrogen recycling while enhancing the metabolic and immune status of the host (Song et al., 2021; Xi et al., 2022). Given that current research predominantly focuses on macroscopic phenotypes, we comprehensively evaluated water quality, growth performance, *Vibrio* counts, gut microbiome dynamics, and transcriptomic responses to investigate the mechanisms underlying these probiotic effects (Chen et al., 2017).

## 2 Materials and methods

### 2.1 Experimental design and rearing conditions

Juvenile Pacific white shrimp (0.33 ± 0.02 g) were obtained and reared at AquaOne Tangshan Aqua-tech Co., Ltd. (39.26 °N, 118.08 °E, Fengnan District, Hebei Province, China). Following a week of acclimation, shrimp were randomly allocated into 18 indoor concrete tanks (6 m × 7 m × 1.5 m; water depth 1 m) at a density of 572 individuals/m^3^. The experimental period lasted for 56 days. Three treatments were established with six replicates in each: N (*P. rettgeri*), NB (*P. rettgeri* + *B. subtilis*, 1:1) NBE (*P. rettgeri* + *B. subtilis* + commercial EM; 1:1:1).

Following standard enterprise protocols, rearing seawater was pre-treated with halogen-based disinfectants, filtered, and kept in the dark for at least 48 h to suppress photosynthetic algae. The rearing tanks were continuously aerated to maintain dissolved oxygen at 5.0–6.0 mg/L, with a daily water exchange rate of approximately 15%. The temperature was maintained at 29 ± 1 °C, salinity at 28.4–29.1‰, and pH at 7.5–8.0. A semi-transparent roof synchronized the photoperiod with natural daylight, supplemented with 1 h of artificial light after sunset. Shrimp were fed a commercial diet (Table 1) five times daily (06:30, 10:30, 14:30, 18:30, and 22:30) at 3–5% of their body weight, adjusted based on feed residues. The input carbon-to-nitrogen (C/N) ratio was controlled at 10:1 (Avnimelech, 1999).

**Table 1:** Proximate composition of the experimental diets (% dry matter).

Nutrients (%)	Proximate composition
Crude fat	7.05
Crude protein	44.84
Crude fiber	3.00
Ash	14.05
Moisture	8.07
Total phosphorus	1.28

### 2.2 Bacterial preparation and supplementation

Single colonies were picked from LB agar plates and inoculated into 2 L LB broth (L8291, Solarbio, China), then incubated overnight with shaking at 37 °C and 150 rpm to reach a concentration of 10^11^ CFU/L. Cultures were harvested by centrifugation at 4 °C, 5,000 rpm for 10 min. Similarly, the commercial EM powder from AquaOne Huizhou Aqua-tech Co., Ltd. was dissolved and standardized to an equivalent concentration of 10^11^ CFU/L. To ensure experimental consistency, the constituent bacterial suspensions were mixed in equal volume proportions (i.e., 1:1 for NB; 1:1:1 for NBE) to achieve a final in-tank density of approximately 10^3^ cells/mL upon application (Li et al., 2024b). Throughout the entire trial, these bacterial solutions were evenly sprayed onto the tank surfaces every 2 days after the last feeding.

### 2.3 Performance indices and sample collection

Daily feed intake and mortality were recorded to calculate the average daily feed intake (ADFI) and survival rate (SR). At the onset and end of the trial, shrimp from per tank were randomly weighed to determine the average daily gain (ADG), feed conversion ratio (FCR), and protein efficiency ratio (PER). At the end of the experiment, hemolymph was collected from the pericardial cavity, clotted overnight at 4 °C, and centrifuged to obtain cell-free serum for biochemical assays. For physiological and molecular analyses, hepatopancreas and midgut tissues were dissected; the former was used for enzyme activity assays and RT-qPCR, while the latter was partitioned for transcriptomic sequencing and RT-qPCR. All tissue samples were immediately frozen in liquid nitrogen and stored at −80 °C. Concurrently, water quality was monitored 24 h post-supplementation for total suspended solids (TSS) and inorganic nitrogen, which are total ammonium nitrogen (TAN), nitrite (NO_2_-N), and nitrate (NO_3_-N) analyses. Bioflocs were concentrated using Imhoff cones and centrifuged (5,000 rpm, 1 min) to collect pellets for microbial community analysis.

### 2.4 Physicochemical properties and *Vibrio* loading of rearing water

The rearing temperature, salinity, and dissolved oxygen were measured daily using a YSI85 multi-parameter instrument (YSI, United States). The inorganic nitrogen contents, including TAN, NO_2_-N, and NO_3_-N, were measured using a Clever Chem automatic discontinuous chemical analyzer (DeChem-Tech, Germany). For *Vibrio* enumeration, water samples were collected from each tank and serially diluted with sterile saline (adjusted to match the rearing salinity). Appropriately diluted samples (100 μL) were spread onto thiosulfate-citrate-bile salts-sucrose (TCBS) agar plates in triplicate. After growing at 30 °C for 24 h, *V. parahaemolyticus* (green colonies) and *V. harveyi* (yellow colonies) were enumerated as pathogenic indicators (Pfeffer et al., 2003). The results were expressed as CFU/mL.

### 2.5 Microbiome analysis

Total genomic DNA was isolated from biofloc samples utilizing the E.Z.N.A.®Soil DNA Kit (Omega Bio-tek, United States) following the manufacturer’s protocols. DNA purity and concentration were verified via 1.0% agarose gel electrophoresis and a NanoDrop 2000 spectrophotometer. The V3-V4 hypervariable region of the 16S rRNA gene was amplified using primers 338F (5′-ACTCCTACGGGAGGCAGCAG-3′) and 806R (5′-GGACTACHVGGGTWTCTAAT-3′). PCR amplification was conducted in a 20 μL volume (4 μL of 5 × Fast Pfu buffer, 2 μL of 2.5 mM dNTPs, 0.8 μL of each 5 μM primer, 0.4 μL of Fast Pfu polymerase, 10 ng of template DNA, and ddH_2_O). Thermal cycling involved an initial denaturation (95 °C, 3 min), 27 cycles (95 °C for 30 s, 55 °C for 30 s, 72 °C for 45 s), and a final extension (72 °C, 10 min). Following purification (PCR Clean-Up Kit, YuHua, China) and quantification (Qubit 4.0), amplicons were sequenced on an Illumina Nextseq 2000 platform. Bioinformatic analysis followed standard pipelines: raw reads were demultiplexed, quality-filtered (fastp v0.23.4; settings: 10 bp sliding window, quality <20, length <50 bp discarded), and merged (FLASH v1.2.7; overlap ≥10 bp, mismatch ratio ≤0.2). Sequences were then clustered into OTUs (97% similarity, UPARSE v11) with chimeric sequences excluded. Taxonomy was assigned via the RDP Classifier (v11.5) against the SILVA database (v138.2, 0.7 confidence). Finally, the OTU table was rarefied to 20,000 sequences per sample, achieving an average Good’s coverage of 99.09%.

### 2.6 Enzymatic activity

The hepatopancreas were homogenized with 0.9% NaCl (w/v). The resulting supernatants were collected for subsequent enzymatic analyses. Activities of amylase (AMY), lysozyme (LYZ), catalase (CAT), and superoxide dismutase (SOD) were determined using commercial kits from Jiancheng (China) with the following catalog numbers: C016-1-1, A050-1-1, A007-1-1, and A001-3-2, respectively.

### 2.7 Real-time quantitative PCR

Total RNA was extracted from the hepatopancreas using TransZol (TransGen Biotech, China) and quantified via NanoDrop 2000 (Thermo Scientific, United States). cDNA was synthesized using the K1621 kit (Thermo Scientific, United States), and RT-qPCR was performed on an Applied Biosystems ViiA 7 system. Target genes included those related to digestion (*lipase*, *lip*; *trypsin*, *try*), lipid metabolism (*peroxisome proliferator-activated receptor γ*, *pparγ*), and innate immunity (*toll-like receptor*, *tlr*; *prophenoloxidase*, *propo*; *anti-lipopolysaccharide factor*, *alf*; *crustin*, *crus*). Primers were designed using Primer Premier 6.0 (Supplementary Table S1). The glyceraldehyde-3-phosphate dehydrogenase (*gapdh*) gene served as the internal control, and relative expression levels were calculated using the 2^−ΔΔCt^ method.

### 2.8 Transcriptome analysis

Midgut tissues were processed for total RNA extraction using RNAiso Plus Reagent (TaKaRa, Japan). RNA quality, including concentration, purity, and integrity, was validated via NanoDrop 2000 spectrophotometry and Agilent 2100 Bioanalyzer analysis. Samples meeting the high-quality criterion (RIN > 7.0) were selected for subsequent library construction.

Sequencing was performed on an Illumina NovaSeq 6000 platform (Majorbio, China) using a 2 × 150 bp paired-end configuration, following standard Illumina protocols. Raw data were subjected to quality control using Trimmomatic. Clean reads were then aligned to the *L. vannamei* reference genome (ASM378908v1) via HISAT2, with transcript assembly conducted through StringTie. Gene expression abundance was normalized as fragments per kilobase of transcript per million mapped reads (FPKM). Differential expression analysis was executed using DESeq2 (v1.24.0), with significant differentially expressed genes (DEGs) defined by a threshold of |log_2_ fold change| ≥ 1 and *p* < 0.05. Finally, the functional implications of these DEGs were explored through the Gene Ontology (GO) and the Kyoto Encyclopedia of Genes and Genomes (KEGG) enrichment analyses.

### 2.9 Statistical analyses

Prior to statistical evaluation, all data were tested for normality and homogeneity of variances. Data were analyzed using one-way analysis of variance (ANOVA) followed by Tukey’s multiple comparisons test via GraphPad Prism 10.0 (GraphPad, United States). Growth performance and survival data are presented as mean ± standard deviation (SD) to reflect individual variation. Other data (e.g., gene expression) are expressed as mean ± standard error of mean (SEM). Statistical significance was defined at *p* < 0.05, with specific levels indicated as *p < 0.05, **p < 0.01, ***p < 0.001, and 0.05 ≤ *p* < 0.1 was considered a trend. In tables, values within the same row without a common superscript lowercase letter indicate significant differences (*p* < 0.05). Bioinformatic analyses for microbiome and transcriptome were conducted using the Majorbio Cloud platform^1^. All omics-related statistical plots were generated using R (v4.2.3).

## 3 Results

### 3.1 Survival rate and growth performance

The effects of progressively complex probiotic consortia on the survival and growth performance of *L. vannamei* are presented in Table 2. Throughout the trial, SR remained comparable across all treatments (*p* > 0.05). However, the dual-probiotic consortium (NB) exhibited superior growth-promoting effects compared to the single-probiotic baseline (N). Specifically, shrimp reared in the NB group achieved the highest ADG and PER, alongside the lowest FCR (*p* < 0.05). Further increasing the microbial complexity did not yield additive benefits. Instead, the triple consortium (NBE) resulted in a decline in feed utilization, evidenced by the lowest PER and the highest FCR among all treatments (*p* < 0.05). Additionally, the ADG in the NBE group reverted to the N baseline level (*p* > 0.05).

**Table 2:** Survival rate and growth performance of *L. vannamei* reared in biofloc systems inoculated with different probiotic consortia (mean ± SD).

Indicator	*N*	NB	NBE	*p*-value
SR, %	76.89 ± 12.17	76.94 ± 13.75	89.04 ± 8.27	0.1953
ADG, g	0.232 ± 0.017^b^	0.258 ± 0.021^a^	0.236 ± 0.020^b^	0.0094
FCR	1.627 ± 0.099^b^	1.470 ± 0.109^c^	1.792 ± 0.134^a^	<0.0001
PER	1.763 ± 0.112^b^	1.954 ± 0.153^a^	1.603 ± 0.121^c^	<0.0001

Different lowercase letters (a, b, c) indicate significant differences among treatments within the same row (*p* < 0.05). Values sharing the same letter are not significantly different.

### 3.2 Water quality and Vibrios loading

The effects of different probiotic consortia on water parameters are illustrated in Figures 1A–C. The dual-probiotic consortium (NB) maintained the lowest concentrations of NH_4_-N and NO_3_-N (*p* < 0.05). It also repressed the NO_2_-N content in comparison with N baseline (*p* < 0.05). The content of TSS rose alongside microbial complexity, reaching a maximum in the NBE group that exceeded the N group (*p* < 0.05). Respecting pathogenic vibrios loading, increased microbial complexity globally inhibited the proliferation of *Vibrio* species (Figure 1D). Both the NB and NBE groups showed a lowering trend in *V. parahaemolyticus* counts compared to the N baseline (*p* < 0.1, respectively). Supplementation of EM (NBE) into the rearing system achieved the minimum *V. harveyi* counts (*p* < 0.05).

**Figure 1:**
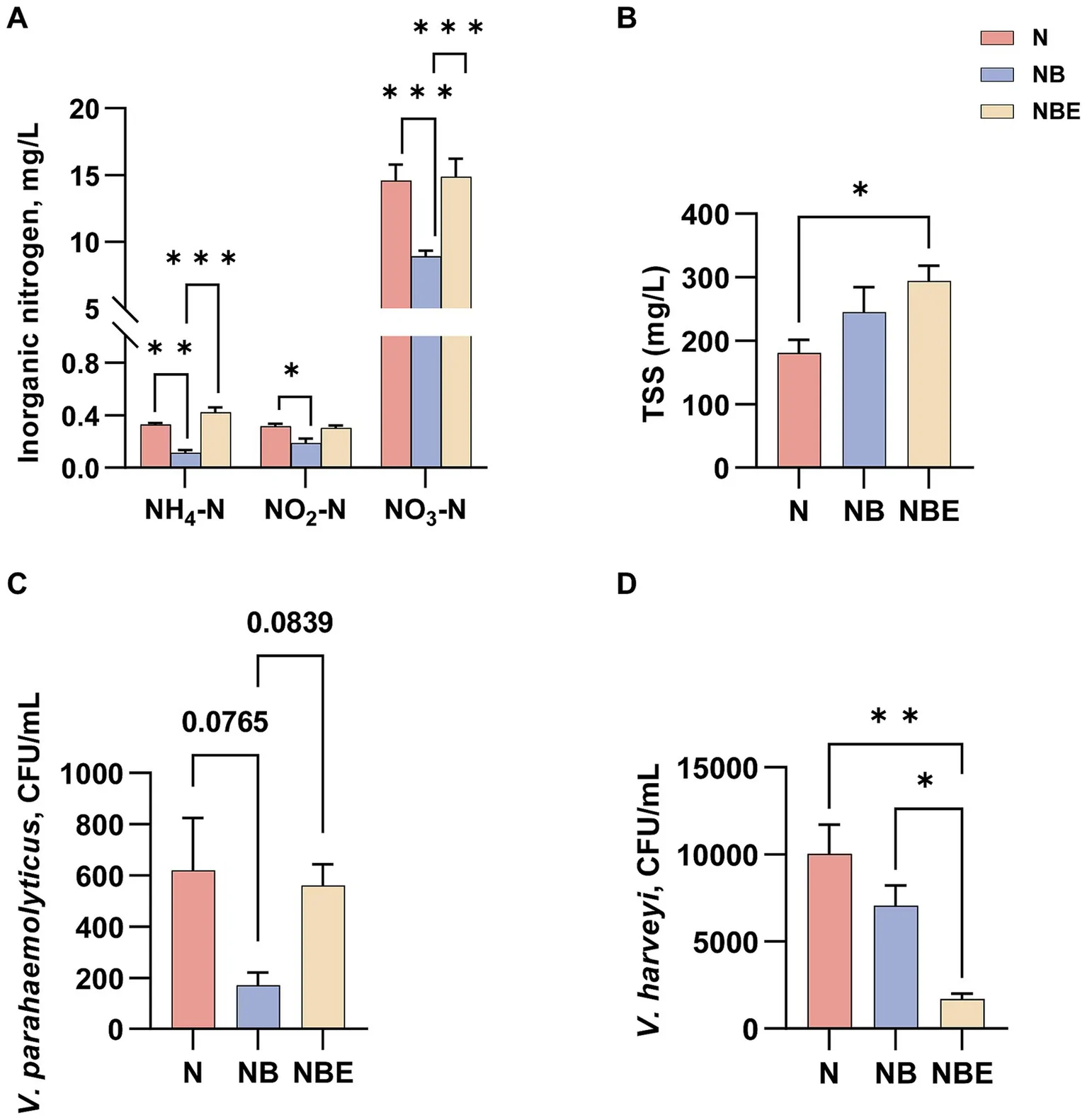
Water quality parameters and pathogenic Vibrio loading in biofloc systems inoculated with different probiotic consortia. **(A)** Concentrations of inorganic nitrogen (NH_4_-N, NO_2_-N, and NO_3_-N); **(B)** Total suspended solids (TSS); **(C)** *V. parahaemolyticus* counts; **(D)** *V. harveyi* counts.

### 3.3 Biofloc microbiome

Both *α*-diversity and *β*-diversity of the biofloc microflora are presented in Figure 2. The ACE, Chao, Shannon, and Simpson indices remained consistent across all treatments (*p* > 0.05). However, the β-diversity demonstrated distinct structural shifts in the microbial communities (*R* = 0.188, *p* = 0.003). In detail, the NB group formed a distinct cluster, whereas the N and NBE groups exhibited partial overlap.

**Figure 2:**
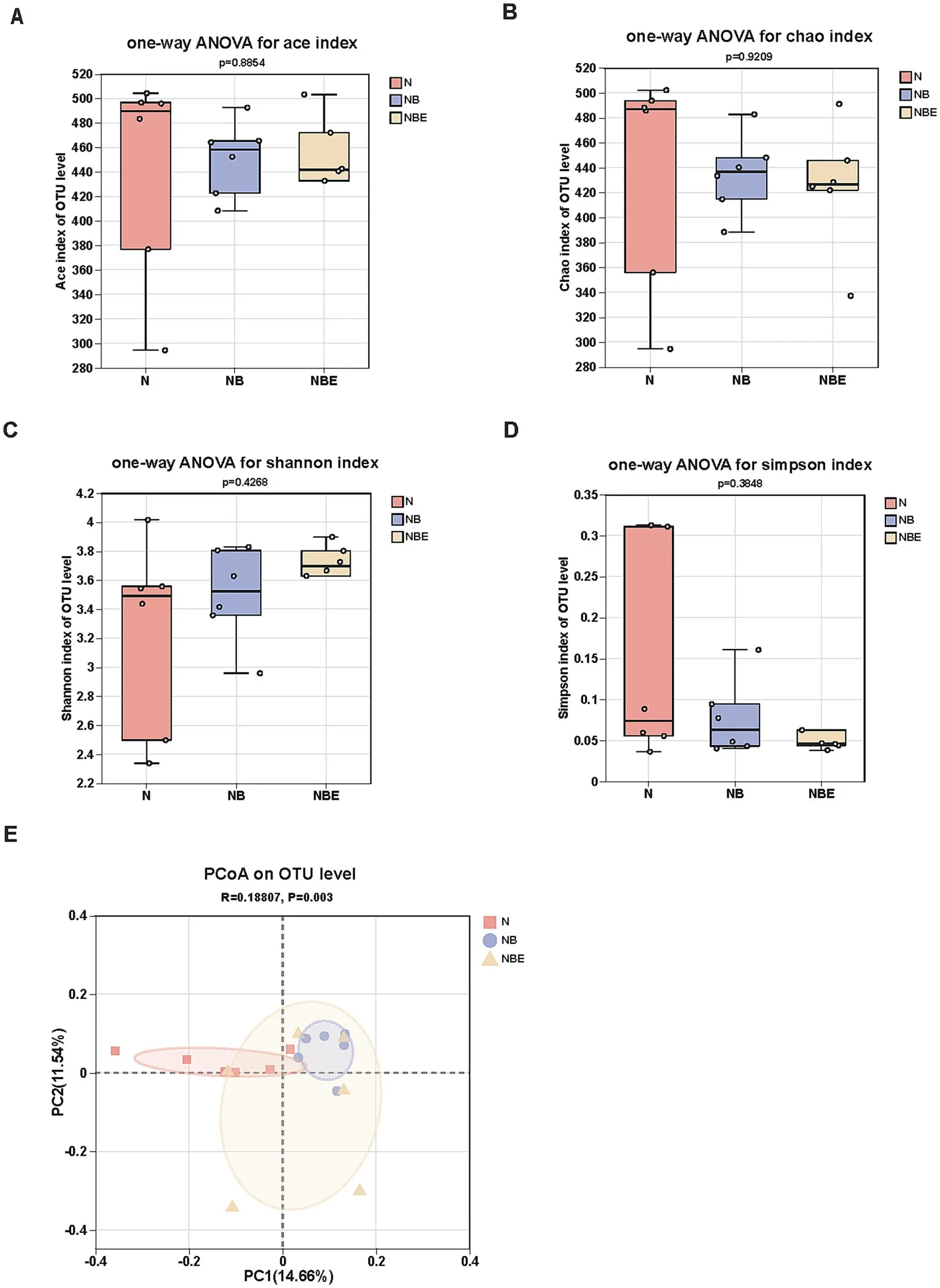
*α* and *β* diversity of the biofloc microbiome at the OTU level. **(A–D)** *α* diversity indices (ACE, Chao, Shannon, and Simpson); **(E)** PCoA based on unweighted unifrac.

Figure 3 reflects the taxonomic composition. N, NB, and NBE groups harbored 85, 13, and 20 unique OTUs, respectively, sharing a core microbiome of 203 OTUs. At the genus level, the relative abundance of *Photobacterium* declined in the NB group, alongside an accumulation of *Vibrio*, *Nitrosomonas*, and *Candidatus Bacilloplasma*. One-way ANOVA confirmed differences in the mean proportions of *Pseudoalteromonas*, Mycoplasmataceae, and *Leuconostoc* across treatments (*p* < 0.05). Furthermore, Rhodobacteraceae, *Ensifer*, and *Clostridium* showed a trend toward differential abundance (*p* < 0.1).

**Figure 3:**
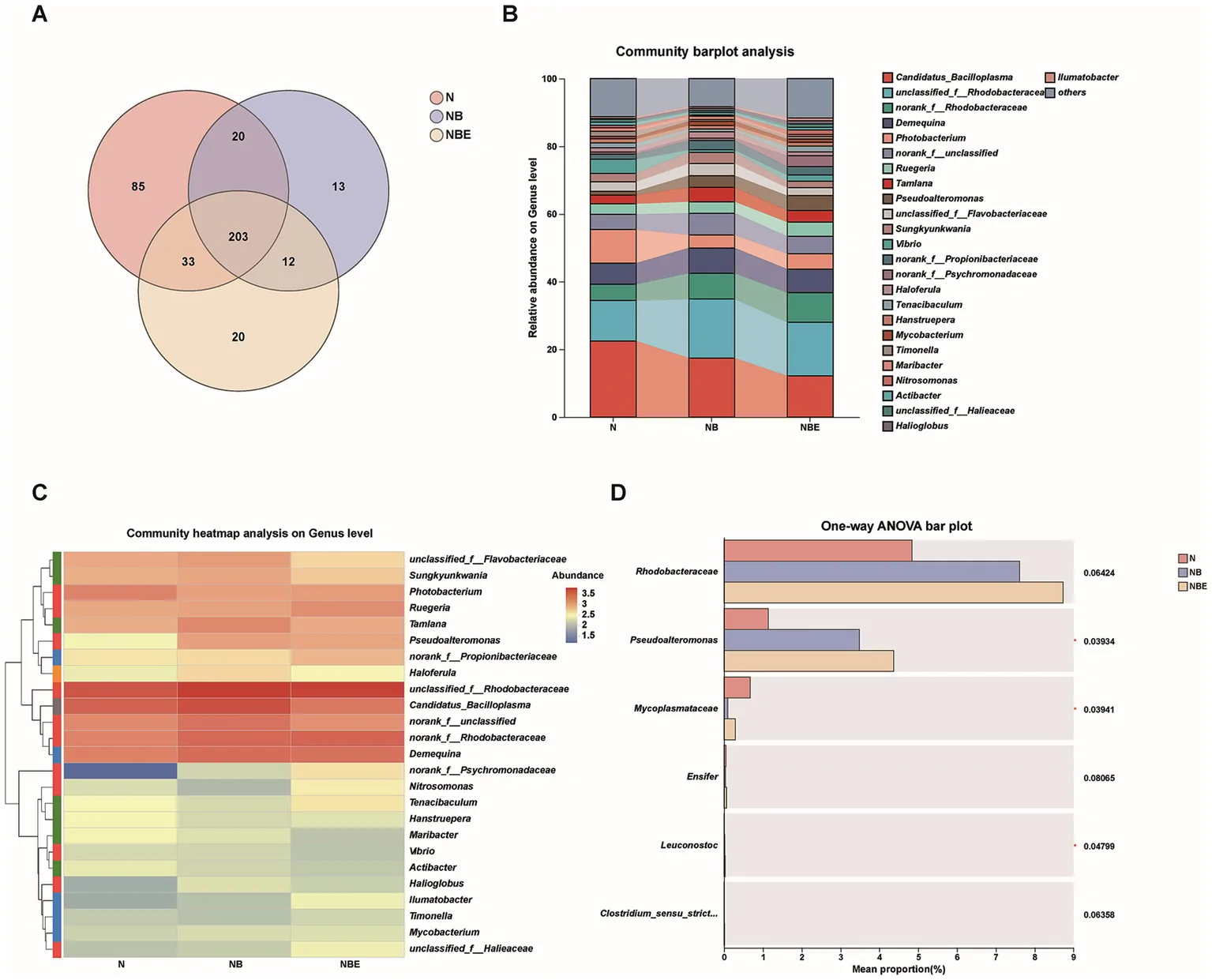
Taxonomic composition of the biofloc microbiome. **(A)** Venn diagram of OTUs; **(B)** Relative abundance of microbial communities at the genus level; **(C)** Heatmap of the 25 dominant genera; **(D)** One-way ANOVA of the top 10 abundant bacterial taxa with significant differences.

### 3.4 Digestive and immune-related enzymatic activities

The effects of probiotic consortia on digestive and immune-related enzymatic activities are presented in Figure 4. Regarding the antioxidant profiles, the dual-probiotic consortium (NB) showed a trend toward increased CAT activity compared to the single-probiotic baseline (N) (*p* < 0.1). Conversely, the triple consortium (NBE) exhibited a trend toward reduced SOD activity relative to the N group (*p* < 0.1). The activities of *α*-amylase and lysozyme did not display statistically annotated differences among the treatments.

**Figure 4:**
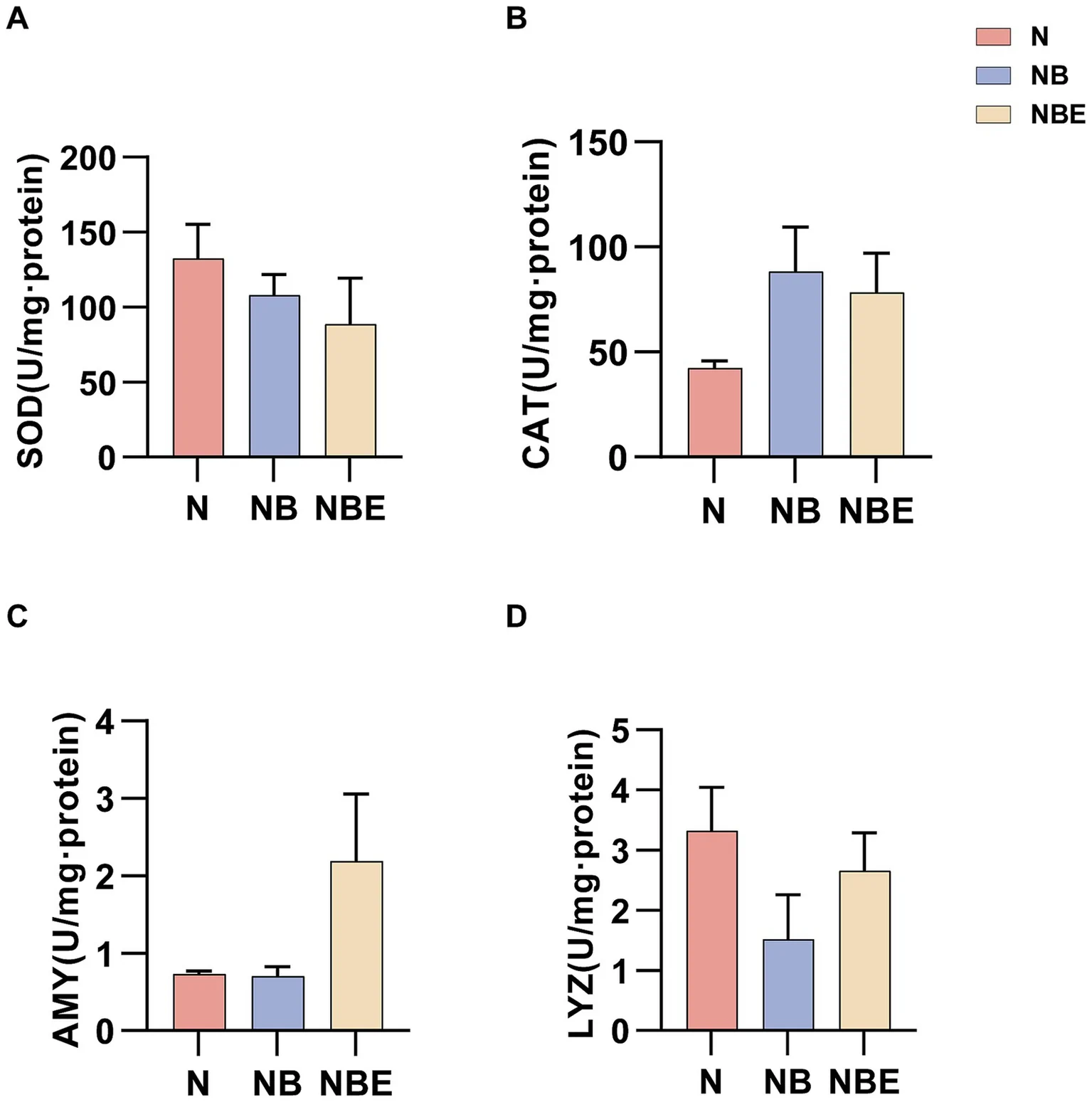
Digestive and immune-related enzymatic activities in the hepatopancreas of *L. vannamei* reared in different biofloc systems. **(A)** Superoxide dismutase, SOD; **(B)** Catalase, CAT; **(C)** α-amylase; **(D)** Lysozyme.

### 3.5 Expression profiles genes relevant in digestion, lipid metabolism, and immunity

The relative mRNA expression of genes related to digestion (*try*, *lip*), lipid metabolism (*pparγ*), and immunity (*tlr*, *propo*, *alf*, *crus*) is presented in Figure 5. The triple consortium (NBE) exhibited the highest levels of digestive genes, with try and lip being upregulated compared with the NB group (*p* < 0.01). Besides, the dual-probiotic consortium (NB) showed higher *try* expression than the N baseline (*p* < 0.01). Furthermore, the transcription level of *pparγ* was upregulated in the NBE group compared to both the N and NB groups (*p* < 0.001). No significant differences were observed in immune-related genes across all groups (*p* > 0.05).

**Figure 5:**
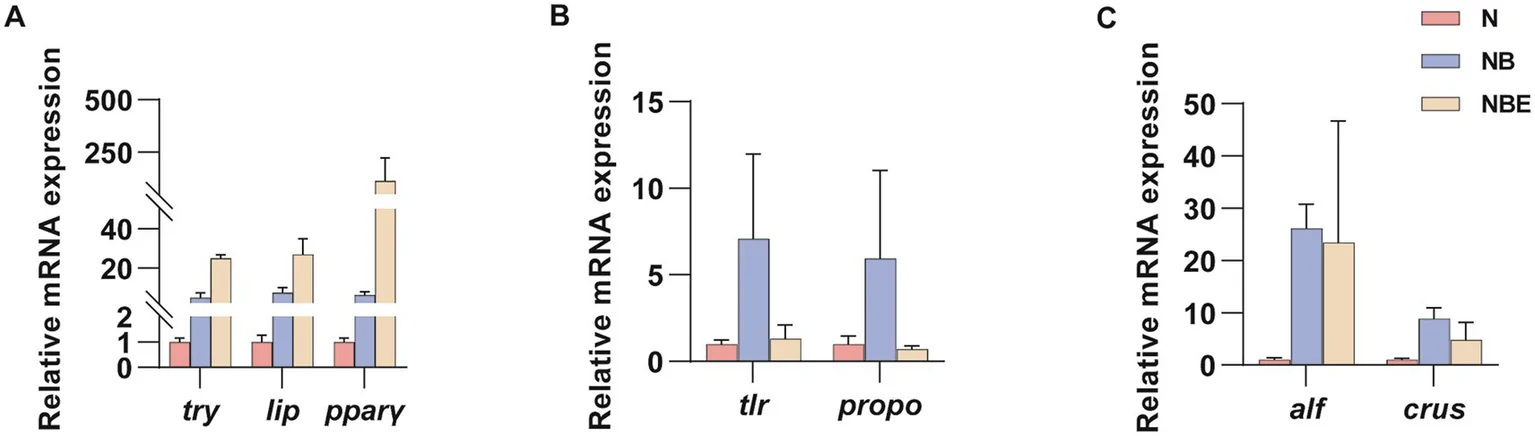
Relative mRNA expression of digestive and immune-related genes in *L. vannamei*. **(A)** Digestion and lipid metabolization (*try*, *lip*, *pparγ*); **(B)** Immune response (*tlr*, *propo*); **(C)** Antimicrobial peptides (*alf*, *crus*).

### 3.6 Midgut transcriptome

The global transcriptomic response in the midgut to different probiotic consortia is shown in Figure 6. PCA revealed distinct clustering patterns (Figure 6A). Specifically, samples from the NB group clustered separately from those of the N and NBE groups, indicating that the dual-strain combination (NB) induced the most pronounced transcriptional reprogramming in the host midgut. Using NB as the baseline, volcano plots illustrate 716 upregulated and 1,218 downregulated genes in N group, 344 upregulated along with 693 downregulated genes in NB group (Figure 6C).

**Figure 6:**
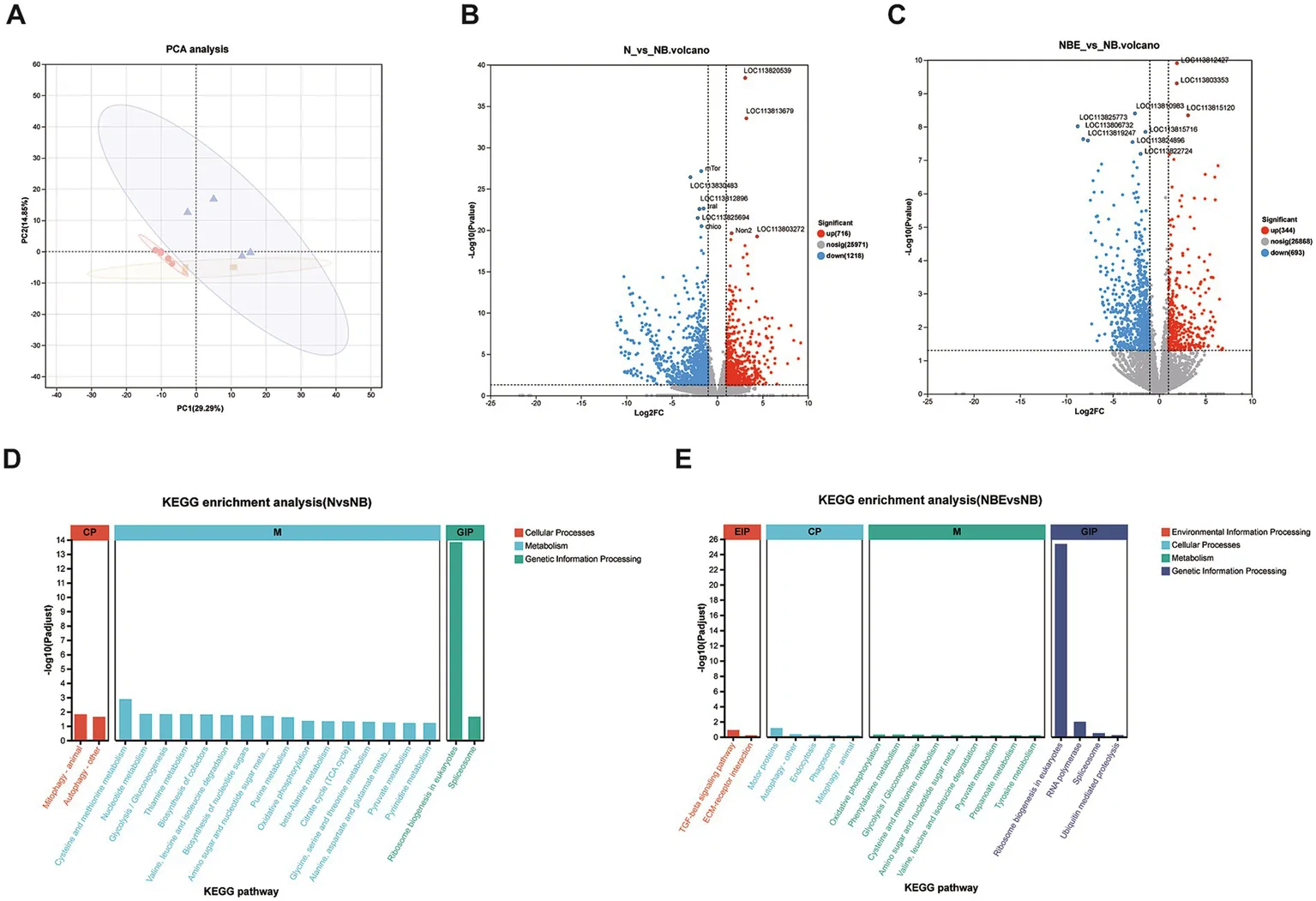
Midgut transcriptomic profiles of *L. vannamei* in response to different probiotic consortia. **(A)** PCA of global gene expression across the N, NB, and NBE groups. **(B,C)** Volcano plots illustrating the differentially expressed genes (DEGs) for the **(B)** N vs. NB and **(C)** NBE vs. NB comparisons. Red and blue dots represent significantly upregulated and downregulated genes, respectively (|log_2_FC| ≥ 1, *p* < 0.05). **(D,E)** Top 20 enriched KEGG pathways of DEGs for the **(D)** N vs. NB and **(E)** NBE vs. NB comparisons.

Pathway enrichment analysis was subsequently performed basing on the KEGG database. In the N vs. NB comparison (Figure 6D), DEGs were enriched in energy and central carbon metabolism pathways, such as the citrate cycle and glycolysis/gluconeogenesis. This finding suggests enhanced aerobic energy production in the NB group. The NBE vs. NB comparison (Figure 6E) revealed distinct enrichments in pathways linking to cellular stress and macromolecular catabolism, exemplified by ubiquitin mediated proteolysis, autophagy, phagosome, and valine, leucine and isoleucine degradation. These transcriptomic profiles indicate that excessive microbial supplementation is even counterproductive. Additional complexity of exogenous microorganisms into biofloc systems triggers a metabolic burden, shifting nutrients toward protein turnover and surveillance pathways.

## 4 Discussion

To the best of our knowledge, this is the first study to systematically evaluate how progressively complex probiotic consortia impact the holistic performance of a biofloc system. Based on our previous findings respecting beneficial impacts of the heterotrophic nitrifying bacterium *P. rettgeri* on shrimp culture (Li et al., 2024a), we expanded the microbial architecture to investigate the phenotypic effects and the underlying ecological and metabolic consequences of multi-probiotic stacking. By integrating growth performance, ammonia and nitrite conversion, and the loading of vibrios, we identified that the dual-probiotic consortium achieved optimal synergistic enhancement. Further increasing microbial complexity by incorporating established commercial EM yielded no additive benefits and unexpectedly deteriorated feed efficiency.

Consistent with classical paradigms in synthetic microbial ecology, these results suggest that negative higher-order interactions frequently emerge in multi-taxa contexts, especially during the assembly of communities from diverse families (Mickalide and Kuehn, 2019). Separately, in biodiversity-ecosystem functioning studies, bacterial services often exhibit saturating responses to species richness under resource limitation (Bell et al., 2005). This saturation is particularly evident in highly constrained environments like biofloc systems, where the microbial community is governed by a strict upper limit of ecological carrying capacity (Emerenciano et al., 2022; Krummenauer et al., 2011). In the current study, while the initial pairing of *P. rettgeri* with *B. subtilis* (NB) likely established effective niche complementarity, increasing consortium complexity in the NBE condition led to pronounced higher-order interactions. These negative higher-order effects are not captured by pairwise additivity, suggesting non-pairwise effects driven by ecological facilitation and behavioral modulation of enzyme expression that ultimately reduce the functional output relative to the best-performing pair (Sanchez-Gorostiaga et al., 2019).

Biofloc systems are inherently microbe-driven and conventionally presumed to be dominated by exogenous microorganisms (Raza et al., 2024). However, our findings, along with some recent reports, pose a challenge to this paradigm. In this opinion, exogenous probiotics rarely achieve absolute numerical dominance within mature bioflocs (Li et al., 2024a). Instead, they function as keystone rare taxa and pioneer colonizers, exerting disproportionately profound influence on community architecture despite their low abundance (Chakraborty et al., 2025; McKenna et al., 2020; Shade and Gilbert, 2015). The effects probably originate from the immediate modification of the microenvironment. By quickly breaking down large organic molecules (Panigrahi et al., 2022) and starting ammonia conversion (Zheng et al., 2025), the dual-probiotic combination set a suitable stage for natively beneficial microbes. On the contrary, the more complex consortia fail to coordinate such synergy. Evidenced by the spike in TSS, their intense metabolic activities will consume excessive oxygen and spatial resources, crowding the environment that ultimately disrupts nutrient cycling and causes the collapse in growth performance (Kim et al., 2022).

The enrichment of beneficially functional bacteria in the NB group further elucidates the holistic impacts of the dual-probiotic combination. Specifically, the proliferation of *Nitrosomonas* aligns with the decline in inorganic nitrogen accumulation, fostering a microenvironment conducive to autotrophic ammonia mitigation (Chen et al., 2023; Robles-Porchas et al., 2020; Caranto et al., 2016). Apart from the processing of nitrogenous substances, the enrichment of Rhodobacteraceae and *Candidatus Bacilloplasma* contributes to host robustness by providing essential enzymes and facilitating a healthy gut microbial community (Simon et al., 2017; Holt et al., 2021). Rather than functioning independently, these beneficial consortia form a complex probiotic-mediated network (Faust and Raes, 2012; Bentzon-Tilia et al., 2016). Such integrated responses collectively drive the observed improvements in FCR and growth performance by simultaneously alleviating environmental stress, enhancing nutrient utilization, and inhibiting pathogens like Photobacterium and vibrios via competitive exclusion (Perry et al., 2020; Knipe et al., 2020). The N group fails to achieve similar growth or health benefits due to limited niche diversity, while the NBE group suppresses beneficial taxa probably through intense heterotrophic competition.

Despite the favorable microflora in bioflocs, the content of pathogenic vibrios (*V. parahaemolyticus* and *V. harveyi*) in rearing water was still considerable. Counintuitively, this situation did not lead to any growth retard or pathological symptoms. This finding highlights the opportunistic nature of this taxon, whose pathogenicity is not inherent but is alternatively triggered by environmental stressors (Defoirdt, 2014; Pilotto et al., 2020). The decent water quality, especially characterized by the low level of nitrite and ammonium, likely eliminated their environmental triggers for virulence expression (Xu et al., 2013). Moreover, the enriched beneficial taxa within the biofloc may also assist to prevent the pathogenic transition through quorum quenching, disrupting their signaling molecules and blocking pathogenic gene expression (Takemura et al., 2014). Furthermore, *Vibrio* may play a functional role by catalyzing floc polysaccharides, which supports their harmonious coexistence within the microecosystem (Chang et al., 2023).

The metabolic reprogramming lends support to the ingestion of biofloc particles and their influence on the host (Chen et al., 2017). The promoted performance in the NB group is originated from the efficient nutrient assimilation. Transcriptomic profiles indicated specific activation towards central carbon and energy metabolism (e.g., TCA cycle and glycolysis), in correspondence to the upregulated digestive genes (*try*) and enhanced antioxidant capacity. These results suggest that the dual-probiotic combination spared more dietary nutrients toward body growth, thereby leading to the observed gains in feed efficiency.

Shrimp in the NBE group are suffering from stress burden as evidenced by the transcriptomic profile in comparison to those in the NB group. Even if the compensatory upregulation of digestive (*try*, *lip*) and lipid metabolism (*pparγ*) genes, these shrimp still exhibited a decline in feed efficiency. This outcome is attributed to the enrichment in macromolecular catabolism and cellular stress pathways, specifically ubiquitin-mediated proteolysis, autophagy, and branched-chain amino acid degradation. As a result, the host shifted to a metabolic trade-off, allocating energy and amino acids away from somatic growth toward cell repair and surveillance (Sokolova et al., 2012; Ciji and Akhtar, 2021). This metabolic burden outweighed the nutritional benefits of enhanced digestion, explaining the impaired growth performance in the NBE group.

To ensure practical relevance, this study was conducted in a super-intensive commercial farm rather than a highly controlled laboratory. Notably, we utilized commercial-scale infrastructure with a standard water depth (1 m). In aquaculture engineering, sufficient water depth is a critical parameter that dictates three-dimensional hydrodynamics, aeration efficiency, and particle suspension. These conditions are difficult to replicate in plastic or glass tanks, which often reach their structural limits and cannot withstand the pressure at such depths (Oca and Masalo, 2013; Gorle et al., 2018). Beyond infrastructure, our findings also challenge the industry trend of broad microbial supplementation. We show that such practices often serve more as psychological reassurance than as a functional necessity. Without precise optimization, these microbial mixes can actually backfire, impairing yields and inflating costs.

Although this study confirms the macroscopic benefits of the dual-probiotic consortium, two critical mechanistic questions remain to be addressed in future research. First, the regulatory pathways of rare taxa within the biofloc microbiome are still unclear. These microorganisms likely modulate community dynamics not through biomass, but via specific signaling molecules—such as acyl-homoserine lactones (AHLs) or autoinducer-2 (AI-2)—which mediate quorum sensing and biofilm formation (Fatimah et al., 2019; Papenfort and Bassler, 2016). Second, the core taxa determining the system output remain elusive. Expanded microbiomic sequencing, coupled with machine learning, will be essential to pinpoint these keystone taxa, thereby enabling the precise evaluation of ongoing biofloc systems (Banerjee et al., 2018).

## 5 Conclusion

Increasing the complexity of exogenous probiotics into the biofloc system does not necessarily promote the output. Our results indicate the dual-probiotic consortium (*P. rettgeri* and *B. subtilis*) outperforms the inclusion of commercial EM in terms of water quality and growth. This advantage originates from the remodeling of microbial community in bioflocs, which enriches beneficial taxa and represses the transition of *Vibrio* to pathogenic state. Excessive supplementation of microbes could result in a metabolic burden, forcing the host to prioritize stress responses instead of growth. Further work should concentrate on optimizing probiotic formulas through more comprehensive trials.

## Data Availability

The transcriptomics data and microbiome data presented in this study can be found in the Genome Sequence Archive (GSA) (https://ngdc.cncb.ac.cn/gsa) under BioProject accession number CRA043666.

## Footnotes

1
https://cloud.majorbio.com
